# Exploring the efficiency of chemically activated palm frond carbon in heavy metal adsorption a modeling approach

**DOI:** 10.1038/s41598-025-89047-4

**Published:** 2025-02-21

**Authors:** W. A. Hammad, M. A. Darweesh, Basant Eweida, M. H. A. Amr, Ahmed Bakr

**Affiliations:** 1 Faculty of Engineering, Tanta University, Tanta, Egypt; 2 Modeling and simulation Research Department, Advanced Technology and New Materials Institute (ATNMRI), City of Scientific Research and Technological Applications, New Borg El-Arab City, Egypt; 3 Tanta Higher institute of engineering and technology, Namlah, Egypt; 4 Environment and Bio-Agriculture, Al-Azhar University, Cairo, Egypt

**Keywords:** Adsorption, Cadmium, Heavy metals, Palm fronds, Wastewater treatment, Mathematical modeling, Environmental sciences, Chemistry, Engineering, Materials science

## Abstract

The industry’s unregulated discharge of unprocessed trash is a major contributor to environmental contamination. Drinking water contaminated by heavy metals is a serious environmental and public health problem. Cadmium (Cd (II)) is one heavy metal frequently found in wastewater. Adsorption is the most widely used method for treating water contaminated with heavy metals due to its great performance, affordability, and ease of use. This work examined the potential for heavy metal adsorption in palm fronds, a chemically treated agricultural waste, using H_3_PO_4_-derived activated Carbon (also known as chemically treated Carbon from palm fronds, or CTCPFs) for Cd(II) adsorption. Extensive batch tests investigated the effects of pH, initial metal concentration, dosage of CTCPFs, and contract duration on adsorption at room Temperature (25 ± 1 °C). Using BET techniques, the surface area and porosity of the sample were measured, and the surface morphology was examined using SEM. FT-IR and XRD measurements were made. After 90 min, 99.65% of the Cd (II) had been removed. Cd (II) was efficiently adsorbed by CTCPFs (> 99%) at pH = 5,C_0_ = 50 mg/l, CTCPFs dosage 1 g /250 ml at 45 ℃ and contact time 90 min. According to the findings, CTCPFs are a cost-effective, efficient, and environmentally friendly adsorbent that can treat heavy metal-contaminated industrial wastewater as impure water. They also show promise in removing Cd (II) under neutral conditions.

## Introduction

Access to clean water is a fundamental human right that people worldwide need and want. Since contamination of pure water supplies impacts living creatures in a permanent or even fatal way, it is a highly contested problem worldwide^1^. One of the major environmental problems attracting increased attention in studies is water contamination^2^. Environmental contamination has gained more attention due to the world’s ongoing technological advancements^3^.

Around the world, there has been a lot of interest in the effects of heavy metal poisoning on aquatic systems and human health. The increasing global population and industrialization contribute to the rising levels of heavy metal pollution in the seas. Another factor causing this issue is the lax enforcement of environmental rules, especially in developing countries. There are two causes of heavy metal pollution: manufactured and natural. Natural causes include flooding, volcanic disasters, and rock degradation. Human activities releasing heavy metals into the environment include residential, commercial, mining, and agricultural sources^4^.

One major concern for the environment is heavy metal pollution. Hazardous heavy metals can build up in the environment and break down through biodegradation^5^. Long-term accumulation of these heavy metals may mitigate the degenerative processes that cause Parkinson’s and Alzheimer’s disease by slowing down the skeletal, muscular, and neurological systems. These heavy metal ions are hazardous and must be removed, both in their elemental form and chemically linked to other molecules^6^.

In comparison to other heavy metals, cadmium (Cd), Copper (Cu ), and Lead (Pb) are extremely toxic and can build up in the body, causing both acute and chronic poisoning in living creatures. Even at extremely low concentrations, cadmium (Cd) harms the body, according to the International Agency for Research on Cancer (IARC). Exposure duration and amount determine the toxic effect of Cd; therefore, prolonged exposure to high levels will result in a more harmful effect. While chronic low-dose exposure to Cd might lead to reduced renal function, a single Cd dosage can cause digestive tract issues.

Cadmium (Cd) easily accumulates in sediments and living things, and it can disrupt biological systems. Organs, including the liver and kidneys, are the targets of cadmium poisoning. In Japan, cases of “lumbago” and two other conditions that can result from cadmium toxicity have also been reported. These illnesses involve bone weakening and cracking.IARC has also designated cadmium as a carcinogenic agent (ICRP)^7^.

The World Health Organization (WHO) states that the maximum amount of cadmium in drinking water is 3 mg L^-1^^8^.

Techniques to extract ions of heavy metals from wastewater include oxidation, coagulation, ion exchange, chemical coagulation, ozone, membrane/nanofiltration, radiation, and biological treatment technologies. However, most of these systems have drawbacks such as high sludge creation, low removal efficiency, high capital investment requirements, high operating costs, and expensive technology. Thus, these methods are inappropriate for small businesses in poor countries. The main obstacle to putting this plan into practice is finding low-cost wastewater treatment technologies to optimize the use of limited water resources and ensure compliance with all health and safety laws about reusing clean wastewater.

Adsorption is a promising alternative therapy because it removes heavy metals. Activated Carbon, zeolite, and clay are popular adsorbents used extensively because of their high adsorption capacity. Studies show that agricultural wastes, previously considered an environmental risk, can now detoxify industrial metal-containing effluents to remove heavy metals. Examples of agricultural trash include bean husks, raw pinecones, sawdust from durian trees, coconut coir, empty oil palm fruit bunches, rice straw, pomelo peel, and palm fronds^9^.

Plant management practices like trimming, fruit harvesting, and rejuvenation include removing the fronds and the plant’s trunk to produce palm oil fronds. Each tree has approximately 60 fronds, each measuring 120 cm^10^.

Egypt has always been a country that grows date palms. In Egypt, the date palm tree is valuable both socially and nutritionally. It has been a significant food source and byproduct for a long time. It is a considerable fruit tree and the most outstanding crop for oasis agriculture due to its ecological advantages. Egypt produces more date palm fruit than any other country in the world. There is a fantastic chance to increase the area where dates are grown to meet domestic needs and create date fruits for export.

Many products and services, including essentials, are made from date palms. Its main product is fruit, which is rich in protein, vitamins, and mineral salts. Because of the yearly pruning, the cultivator can utilize the secondary products of the palm^11^.

Since each of Egypt’s 17 million palm trees yields more than 25 kg of fruit a year, palm leaves are regarded as a significant agricultural residue produced in Egypt and the Arab globe each year. Rich in cellulose, carbohydrates, and other essential components, these field wastes can be used in various industries or to recover precious natural resources rather than being burned^12^.

Mathematical Modeling and simulation have drawn interest recently as a method with a developing impact on diligent output. These methods make it possible to create mathematical equations from the data obtained from A small sample of experimental findings that can be used to model and simulate, saving time and money. Response Surface Methodology (RSM) makes it simple to associate variables with one another, making it ideal for enhancing various quadratic polynomial model processes. The Box-Behnken factorial design (BBD) in a conventional behavior was the foundation for the adsorption processes that the RSM investigation examined. Using activated Carbon from a palm frond, nearly all parameter tests were conducted individually to investigate the degrees of the elements that impacted the Cd (II) percentage removal (%). These variables were time (min), concentration (mg/L), and dose (mg). One benefit of RSM is that it requires fewer experiments, which means that more expensive analytic methods are unnecessary. This method can be used for the BBD or the CCD^13^.

Water quality and human health are seriously threatened by the growing environmental contamination caused by uncontrolled industrial discharge, especially when heavy metals like cadmium (Cd (II)) are present in wastewater. The potential of employing H_3_PO_4_-treated palm frond carbon (CTCPFs) as an affordable and environmentally friendly adsorbent for the removal of Cd (II) is investigated in this work. The effects of adsorbent dosage, pH, initial Cd (II) concentration, and contact time on adsorption effectiveness were systematically assessed at ambient temperature (25 ± 1 °C) using a series of batch tests. In order to evaluate the surface area, porosity, and shape, the material was characterized using BET analysis, SEM, FT-IR, and XRD techniques. The findings showed that CTCPFs were an effective adsorbent, with a high Cd (II) removal rate of 99.65% in 90 min. According to the results, CTCPFs are an economical and environmentally beneficial way to treat heavy metal-contaminated industrial wastewater. They are especially good at removing Cd (II) in neutral conditions.

## Materials and methods

### Adsorbent preparation

Palm frond samples were sectioned into intervals of one to three centimeters. The objects were thoroughly cleaned to remove the impurities, dust, and fibers. Tap water was used initially; then distilled water was washed. To remove moisture, samples of palm fronds were dried in a Lenton oven at 105 °C for five hours. The powdered dehydrated palm fronds (DPFs) were obtained. A 60% H3PO4 solution was used to soak 5 g of the powdered material to create activated Carbon (AC). After combining the oil palm fronds, 15 milliliters of 60% H3PO4 solution were weighed. After two hours in a muffle boiler at 100 °C for impregnation, the samples were activated for three hours at 400 °C. The sample was washed to get rid of the activated Carbon’s acidic component. Washing was done constantly up until pH 7. to remove any remaining moisture. Then, the samples were roasted at 110 °C to dry them completely.this process enhances the porosity, surface area, and functional groups of the activated carbon. These properties are critical for applications like adsorption of heavy metals, as they determine the material’s ability to interact with and retain target molecules or ions.

### Preparation of Cd(II) stock solution

Analytical quality chemicals were consistently used in all tests. One liter of deionized water was used to dissolve 2.036 g of Annular-grade cadmium chloride to create the stock solution, which had a concentration of 1000 mg Cd (II)/L. Dilution was then used to create working solutions with different starting concentrations. The solutions’ initial pH was raised using 0.1 mol/L of HCl and 0.1 mol/L of NaOH.

### Adsorption experiment

**Fig. 1:**
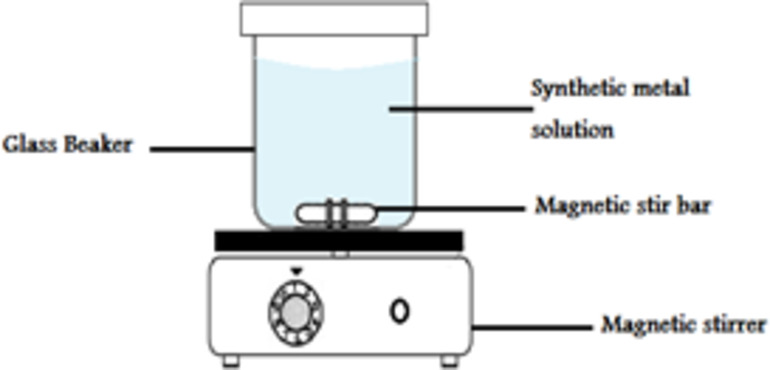
The apparatus used for Cd (II) adsorption.

**Fig. 2:**
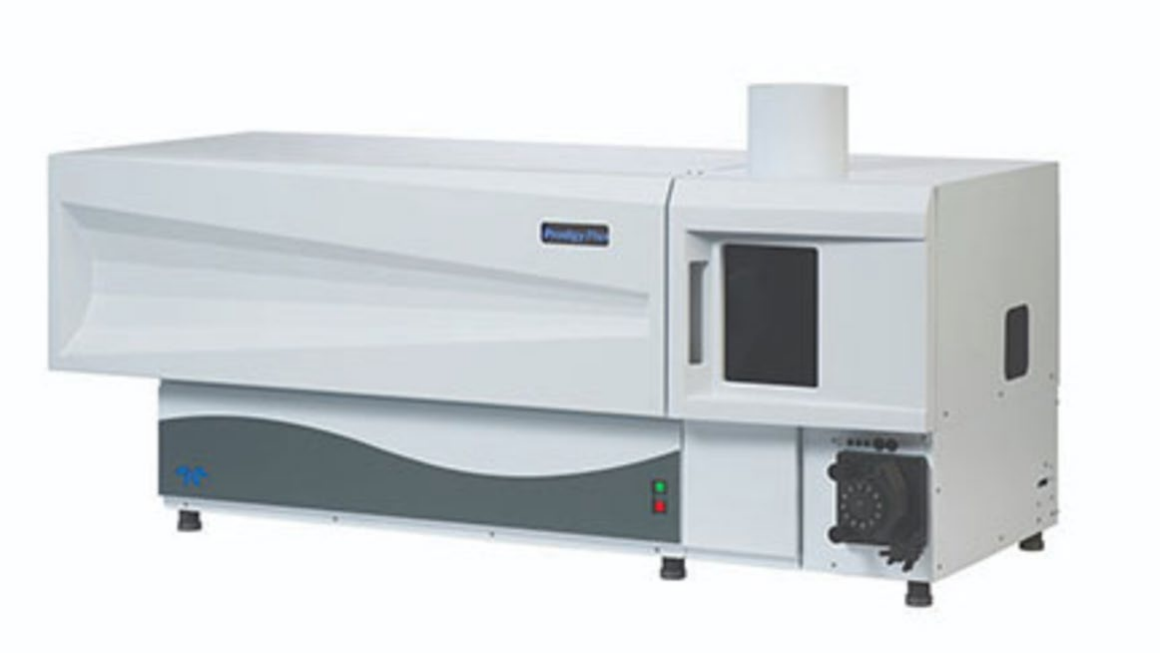
Inductively coupled plasma.

Studies for Cd (II) removal over CTCPFs in 250 ml conical flasks were conducted using the batch method. The initial concentration of Cd (II) (Co) varied between 50 and 300 mg/L; the contact period varied between 5 and 90 min; the pH ranged between 2 and 8; the adsorbent dose (m) varied between 0.1 and 1 g; and the temperature (T) varied between 20 and 45 °C. The pH of the initial solution was adjusted using either 0.1 M HCl or NaOH solution. The Cd(II) solution was combined with a ceramic hot plate stirrer (Model C230V50/60Hz) at 3000 rpm in order to reach equilibrium, as shown in Fig. 1. A beaker holding an adequate amount of adsorbents was then filled. After the predetermined amount of time had elapsed, 5 ml of the Solution was obtained as a test sample, and it was centrifuged (Model C230V50/60Hz) for 15 min at 4000 rpm to extract the liquid phase’s adsorbents. The supernatant was stored for use in metal concentration studies using Prodigy Plus ICP-OES inductively coupled plasma-optical emission spectrometry (ICAP 6000). Fig. 2.

To compute the percentage of Cd (II) elimination (%), Eq. 1 was used

1 $$\:\varvec{\%}\:\varvec{R}\varvec{e}\varvec{m}\varvec{o}\varvec{v}\varvec{a}\varvec{l}=\mathbf{\%}\:\mathbf{a}\mathbf{d}\mathbf{s}\mathbf{o}\mathbf{r}\mathbf{p}\mathbf{t}\mathbf{i}\mathbf{o}\mathbf{n}=\left(\frac{{\varvec{C}}_{0}-{\varvec{C}}_{\varvec{t}}}{{\varvec{C}}_{0}}\right)\times\:100$$

Where:

The initial concentration of Cd (II) in the Solution at time t (mg/L) is represented by C_0_, and Ct represents the equilibrium concentration.

### Activated carbon preparation’s characteristics

Using BELSORP max II equipment from Japan, the surface area and porosity of the produced sample were determined from N_2_ adsorption measured at -176℃. First, the artwork was outgassed at 100 °C for a full day using a 10 − 4 Torr Hoover cleaner. After the pressure reaches saturation, all pores are liquid-filled, making assessing the sample’s fine pore structure feasible. The condensed gas was extracted from the system by progressively decreasing the adsorptive gas pressure. The information was used to connect the adsorption and desorption isotherms. The adsorption isotherm displays the adsorbed amount as a function of pressure at a certain temperature. The Brunauer-Emmett-Teller (BET) method calculates the specific surface area^14^. Computing the microporous volume and porous structure distribution is common practice using the Barnett-Joyner-Halenda (BJH) technique.

To examine the surface morphology of the materials, X-ray analysis, and SEM-EDX were evaluated as a supplement to wet chemical analysis. Using a VEGA II LMU (Tescan) scanning electron microscope outfitted with INCA Energy 450/XT (Silicon Drift detector, SDD) and INCA Wave 700 (crystals: LiF, PET, TAP, LSM60, LSM200) EDS equipment, the micro-texture and elemental composition of the samples were evaluated. The INCA Energy Plus software suite allows for the simultaneous usage of both systems. The BSE detector was used to create the SEM images. Perkin Elmer Spectrum 2 picked up the FT-IR spectrum. The powder was subjected to X-ray pattern analysis using a PW3040/60 Analytical Diffractometer.

## Results and discussion

### Activated carbon preparation characteristics

**Fig. 3:**
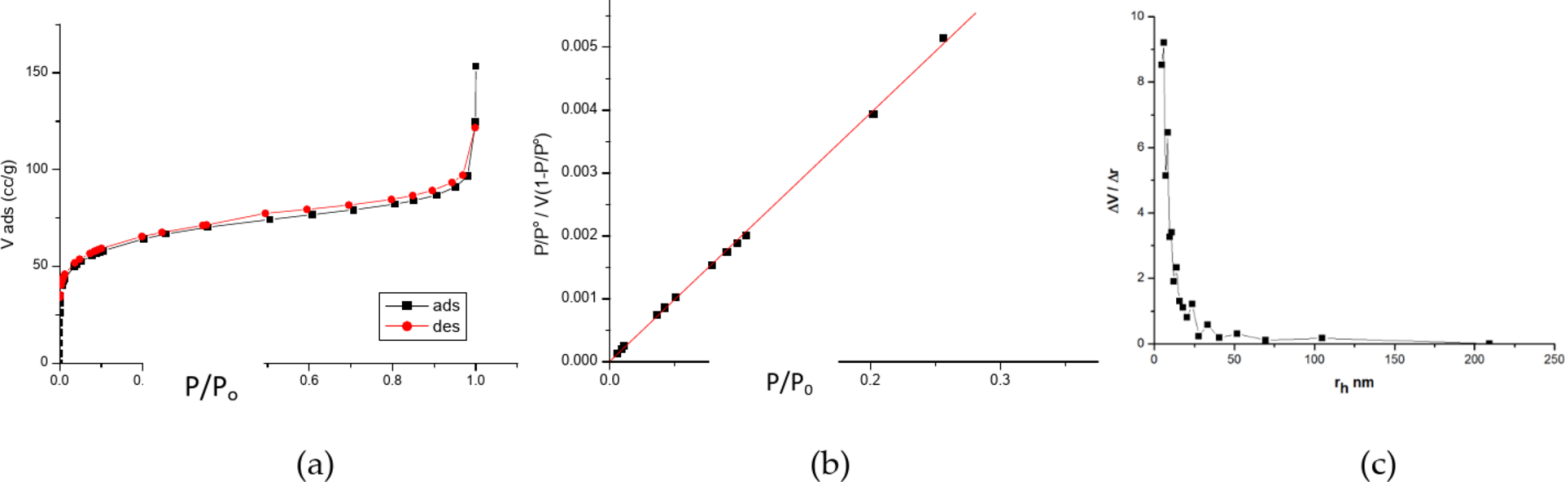
(**a**) The N_2_ adsorption-desorption isotherm prior to the adsorption processes, (**b**) BET surface area plot before adsorption. (**c**) Pore size distribution by BJH before adsorption.

The nitrogen adsorption-desorption isotherm plot of oil palm fronds before adsorption is displayed in Fig. 3(a). The hysteresis loop indicates the isotherm and its smooth First-order capillary condensation transition. Microspores and a type II isotherm are visible in the isotherm. Monolayer formation is correlated with the flat area at low pressure. Mesopores, or microscopic holes, are indicated by the closure of the hysteresis loops. The isotherm also displays a type H3 hysteresis loop, which is explained by the presence of interparticle mesopores created when small crystallites aggregate.

Evaluations of the external and pore areas are considered to compute the total surface area in m^2^/g. The BET equation is commonly written as follows.

2 $$\:\frac{\raisebox{1ex}{$P$}\!\left/\:\!\raisebox{-1ex}{${P}_{0}$}\right.}{v\left[1-\left(\raisebox{1ex}{$P$}\!\left/\:\!\raisebox{-1ex}{${P}_{0}$}\right.\right)\right]}=\frac{c-1}{{v}_{m}c}\left(\frac{P}{{P}_{0}}\right)+\frac{1}{{v}_{m}c}\:$$

The quantities of adsorbed gas (v) and monolayer adsorbed gas (v_m_) are represented by the values P and P_0_, respectively, at the adsorption temperature; the BET constant is defined by the value c.

The experiments’ findings indicate that Eq. (2) represents an adsorption isotherm. And can be shown as a straight line with P/P_o_ on the x-axis and 1/v(1-P/P_o_) on the y-axis. This plot is a BET narrative. The linear relationship of this Equation is only maintained in the 0.04 P/P_o_ 0.45. With the values of the line’s slope (c-1/v_m_ c) and intercept (1/v_m_ c), one can determine the monolayer adsorbed gas quantity v_m_ and the BET constant c. Next, the mean pore radius (r), total pore volume (V_t_), and specific surface area (SBET) were computed. The following summary of the findings: V_t_=96.290 cc/g; *r* = 2.37 nm; v_m_=85.1 cc/g; c = 1.02; SBET = 340.86 m^2^/g. Figure 3(b).

The Barret, Joyner, and Halenda pore size model (BJH) was created to compute the PSD over the mesopore and partial micropore range. BJH pore dimension

3 $$\:{\:\:\:\:\:\:\:\:\:\:\:\:\:\:\:\:\:\:\:\:\:\:\:\:\:\:r}_{p}={r}_{k}+t$$

Where t is the thickness of the adsorbed layer, r_p_ is the pore’s actual radius, and r_K_ is the pore’s Kelvin radius.

The statistical thickness reduction of the pores during desorption is known as BJH. Figure 3 (c) shows the sample’s pore size distributions as ascertained by the BJH method and the desorption branches of isotherms. The micropore area shows the distribution of microscopic pore diameters. Based on the surface area and pore volume measurements, the average pore diameter is 2.398 nm.

**Fig. 4:**
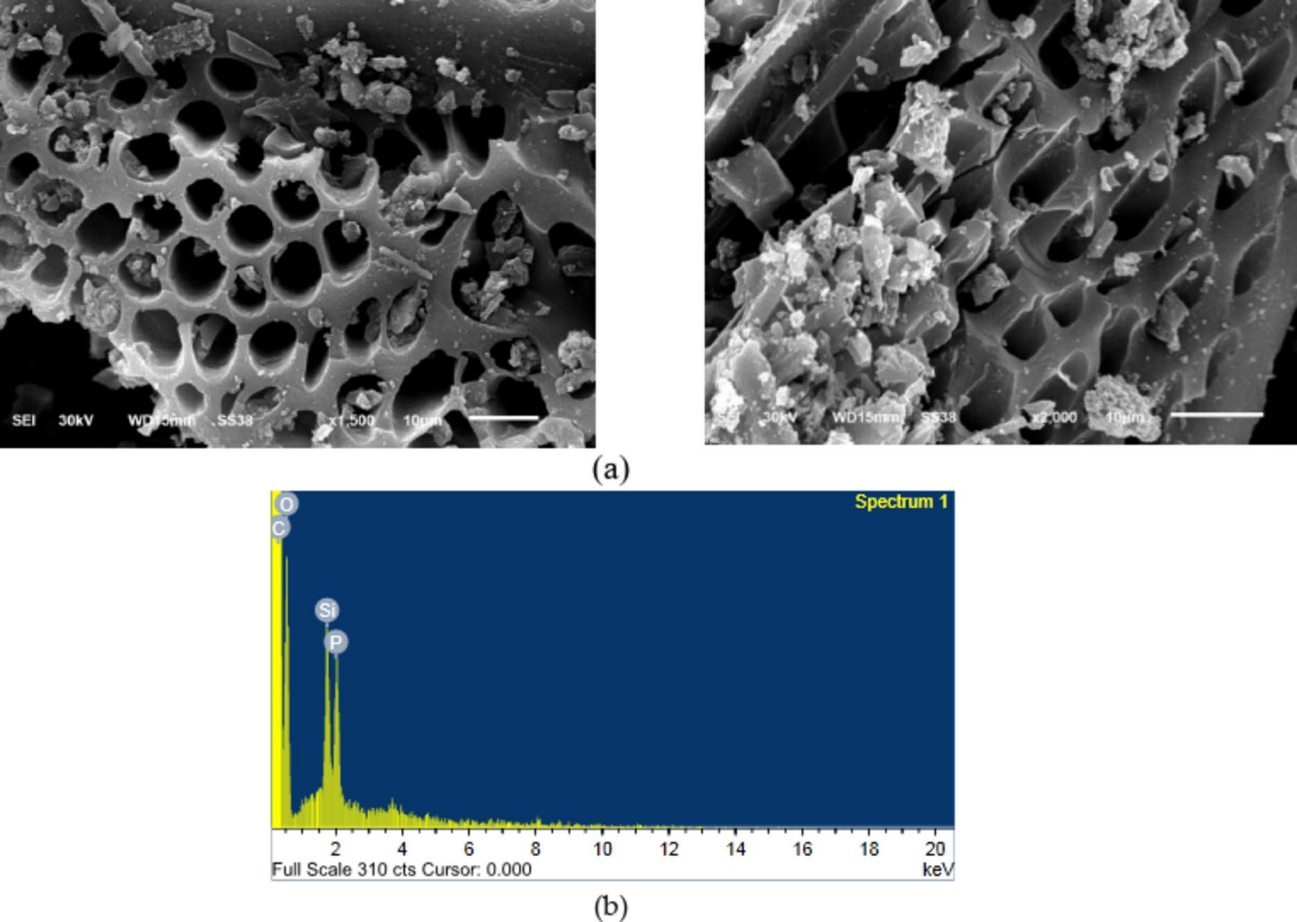

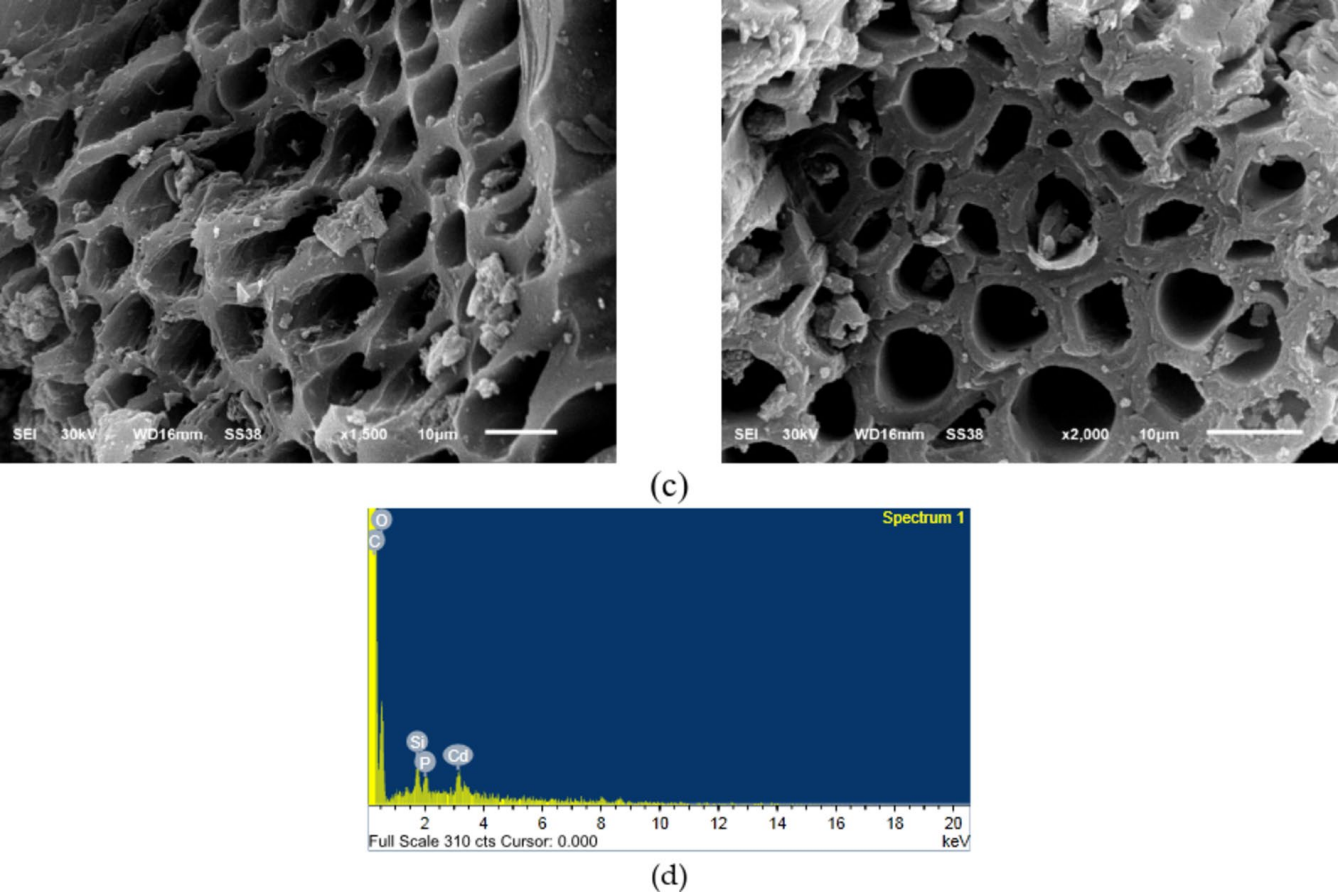
SEM images of CTCPFs prior to (**a**) and (**b**) EDX analysis of CTCPFs. And SEM images of CTCPFs after Cd (II) adsorption onto CTCPFs (**c**). (**d**) EDX analysis of Cd (II) absorbed onto CTCPFs.

Figure 4a, c shows SEM images of AC samples before and after Cd (II) removal (CTCPFs /Cd (II)). The topography of the surface agrees with the surface area and porosity values determined by BET and BJH measurements. The SEM-EDX analysis results, shown in Fig. 4 (b) and( d) of the sample adsorbed Cd (II), verified the presence of Cd (II) compound at a 2-weight% concentration.

**Fig. 5:**
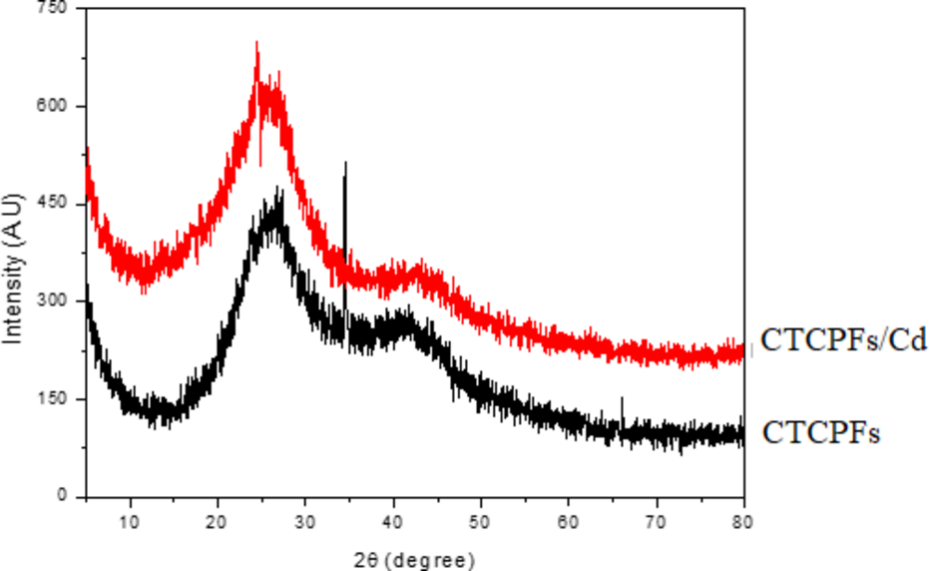
XRD pattern of CTCPFs, and Cd (II) adsorbed on CTCPFs.

The XRD pattern recorded for Cd (II) adsorbed on CTCPFs in palm fronds is displayed in Fig. 5. Samples are exposed to radiation from Cd. Ka = 1.54, with a scanning range of 2 and a range of 5 to 80. Adsorption of Cd (II) on CTCPFs results in a diffraction pattern with a large peak at 2 θ = 26, indicating the amorphous character of the material^15^. There is also no organized crystalline structure, as the observed XRD pattern demonstrates.

**Fig. 6:**
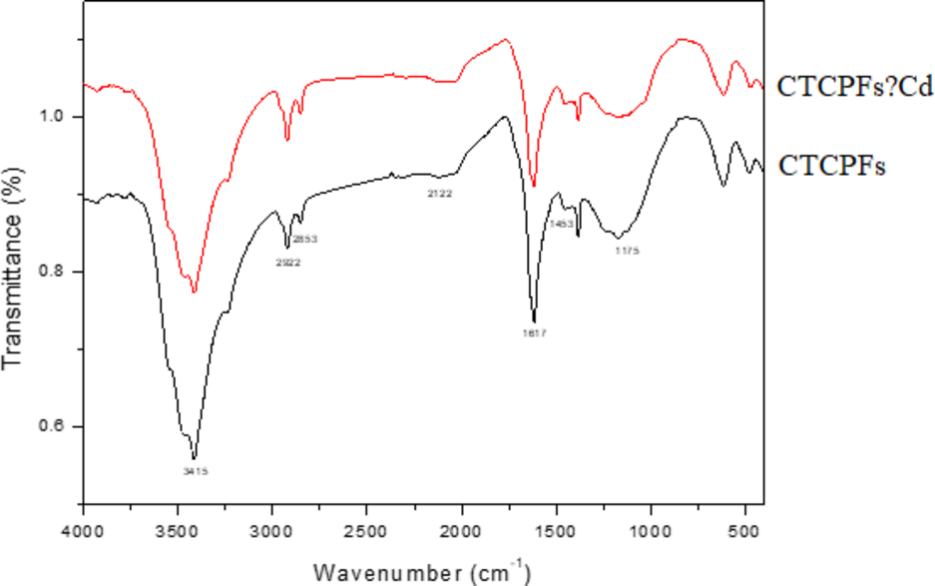
FT‒IR spectra of CTCPFs and Cd (II) adsorbed on CTCPFs.

The FT-IR (KBr, cm^-1^) of Phoenix dactylifera CTCPFs is shown in Fig. 6 following burning at the following wavelengths: 1453 cm^-1^ (C = C stretch), 1175 cm^-1^ (C-H bending vibration), 1617 cm^-1^ (C = O stretching of acid), 2922 and 2853 cm^-1^ (CH-from. ), and 2122 cm^-1^ (CH-aliphatic. FTIR spectrum data show that CTCPFs (Phoenix dactylifera) contain flavonoids and polyphenols.

**Fig. 7:**
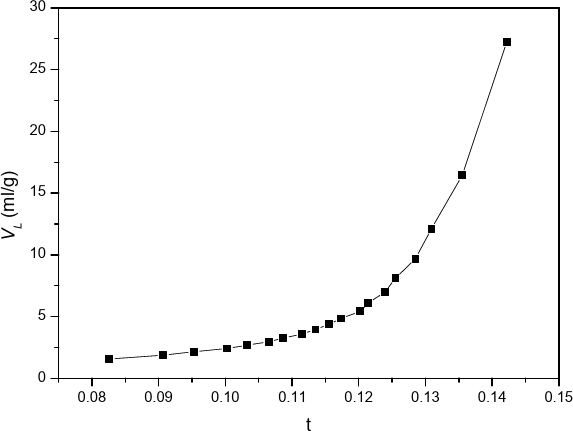
t-plots before adsorption.

The experimental data was processed using the t-plot method to determine the exterior surface area, micropore volume, and total pore volume. Layer thickness “t” was calculated as a function of increasing p/po using a mathematical model of the multilayer. As pressure rises, the adsorbate layer’s thickness (“t”) also rises. Figure 7 shows the experimental volume adsorbed (V_L_) versus statistical thickness (t) for each p/p_o_. Lippens and de Boer’s classification states that the sample exhibits an upward deviation, suggesting meso-porosity. The SBET value and the surface area St(m^2^/g) were determined by calculating the slope of the straight lines that run through the origin. Fig. 7^16^.

### Effect of pH

**Fig. 8:**
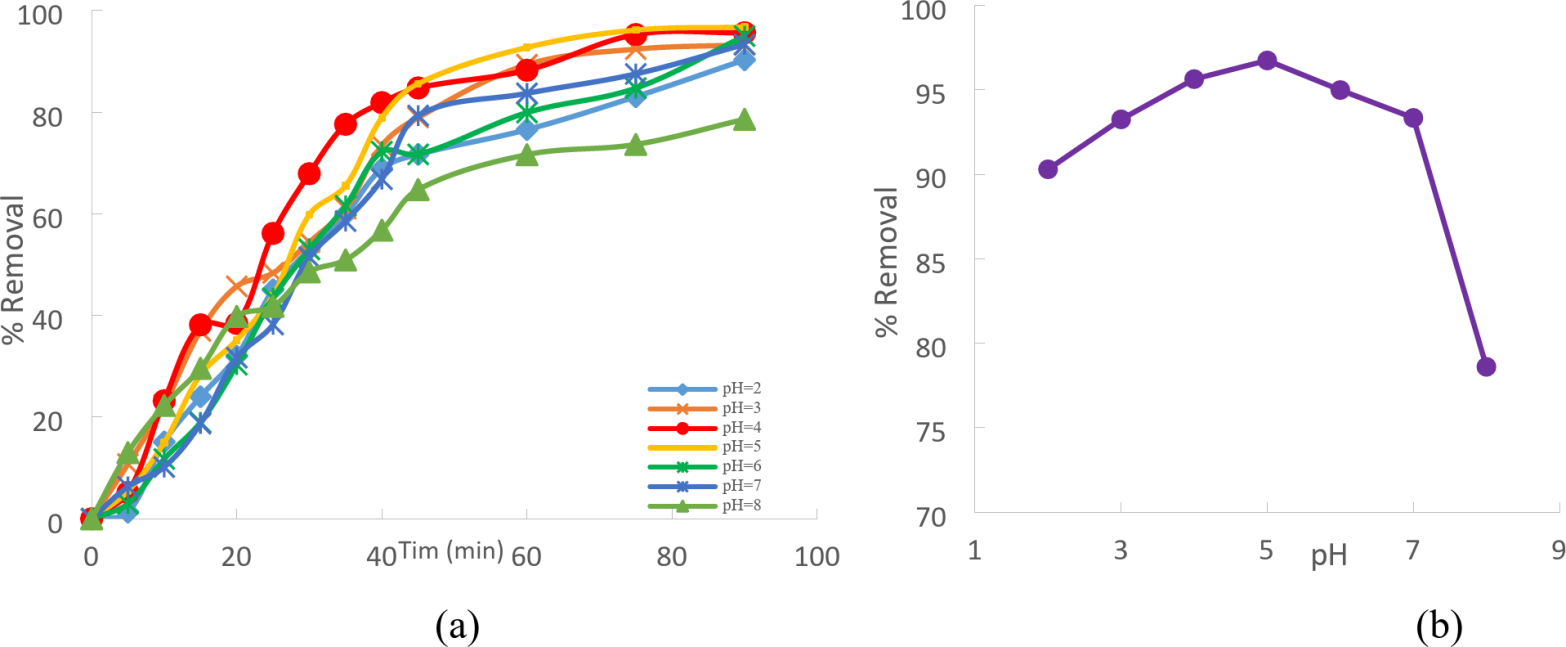
(**a**) pH effect regarding Cd (II) adsorption onto CTCPFs (initial concentration = 50 mg/L, stirring speed = 300 rpm, CTCPFs dose = 0.5 g/250 ml T = 25 ℃ and time = 90 min). (**b**) pH effect on Cd (II) removal yield.

PH is an essential adsorption property for Cd (II) removal from aqueous solutions. It impacts the adsorbate parameters, ionization level, and surface charges. To attain complete equilibrium, the initial Cd (II) concentration and the adsorption contact time are 50 mg/L and 90 min, respectively. Understands how pH affects Cd (II) absorption and conducts equilibrium tests at different pH levels. Between two and eight are employed. This makes sense, given that Cd (II) ions tend to produce precipitation at higher pH values^17^. pH 211 HANNA - Romania The pH of the liquids was adjusted using a pH meter, Fig. 8a.

The percentage removal will rise as pH rises, up to pH = 5. The ability to trap metal ions in extremely acidic media was restricted and progressively increased with increasing pH levels before declining when the medium’s pH exceeded a particular value, or 5^18^. As the pH rose, Cd(II) cations did not always compete with H^+^ for adsorption sites, favoring the deprotonation process and increasing Cd(II) uptake. When the pH of the Solution was higher than 5.0, Cd(OH)^+^ was the Cd(II) ion that CTCPFs adsorbed the least readily, which resulted in a little decrease in the amount of Cd(II) that could be absorbed^19^.percentage removal of Cd^+ 2^ increases from 90.29% to96. 74%. For pH values from 2 to 5 respectively, increasing pH value will decrease the percentage removal of Cd^+ 2^ for pH values from 6 to 8. Figure (8 b).

### Effect of initial concentration

**Fig. 9:**
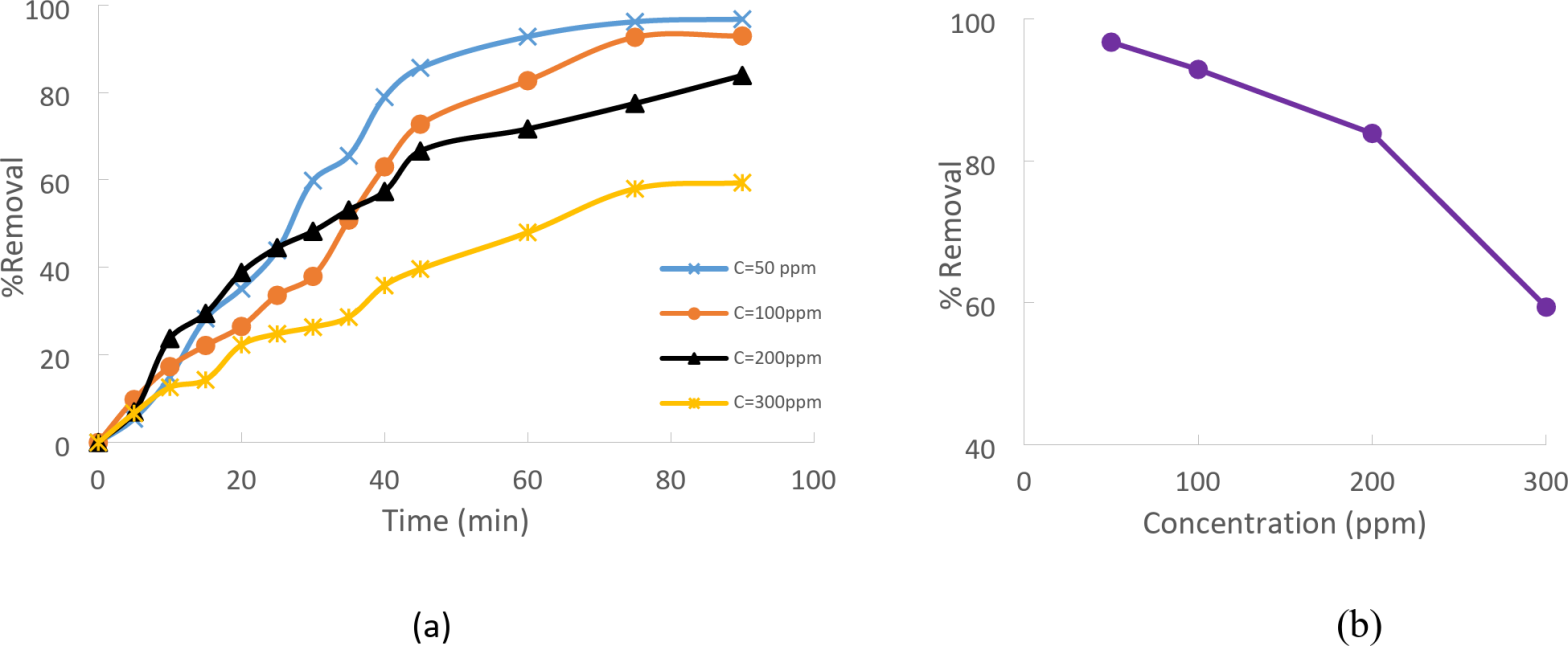
(**a**) Effect Cd (II) initial concentration adsorption onto CTCPFs (pH = 5, stirring speed = 300 rpm, CTCPFs dose = 0.5 g/250 ml, T = 25℃ and time = 90 min). (**b**) Effect of Cd (II) initial concentration on adsorption.

The impact of Cd(II) starting concentrations on the percentage of Cd(II) removed from CTCPFs was investigated at a temperature of 25˚C, with a contact period of 90 min and a pH of 5. Additionally, the stirring speed was 300 rpm. For varying Cd(II) C_0_ (50, 100, 200, and 300 mg/L), the CTCPFs dose equals 0.5 g/250 ml. According to Fig. (9 a), when the concentration of Cd (II) has increased, the percentage clearance of Cd (II) has decreased. When the concentration is raised, the empty spaces on the adsorbent surface where Cd^+ 2^ is adsorbed at low concentrations become saturated and filled. Many active sites on the adsorbent’s surface are open to heavy metal adsorption when the concentration of Cd^+ 2^ is low. On the other hand, if the initial Cd^+ 2^ concentration is raised, there will be more Cd^+ 2^ moles than empty sites. As a result, the metal ions clearance rate drops, and the accessible sites are rapidly saturated^20,21^. Consequently, C_0_ of Cd (II) increased from 50 parts per million to 300 parts per million, even though the percentage of Cd (II) removed fell from 96.74 to 59.38%^22^. Figure 9(b) .

### Effect of adsorbent dose

**Fig. 10:**
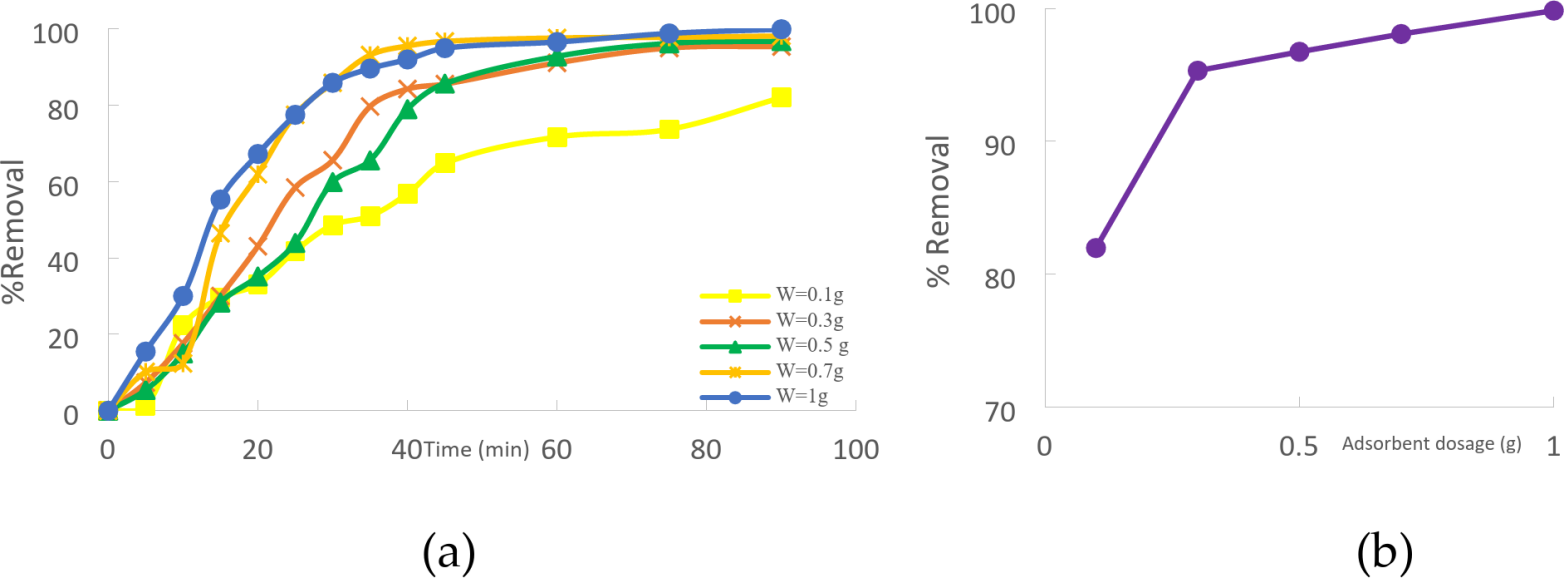
(**a**) Effect of CTCPFs dose on % removal Cd (II) with C_0_ = 50 mg/L, contact time 90 min, stirring speed = 3000 rpm, T = 25 ˚C and pH = 5. (**b**) Effect of CTCPFs dose Cd (II) removal.

The effect of adsorbent dosage on removing Cd(II) took 90 min for a starting concentration of 50 mg/L. Various dosages of adsorbents (CTCPFs) = (0.1, 0.3, 0.5, 0.7, and 1 g/250 ml. Figure 10a shows how the % Cd(II) removed increases with the height at which the amount of adsorbent is used. A high concentration of CTCPFs during the adsorption process can ensure the availability of more sites and particular surface areas for adsorption, which often results in a high adsorption capacity. An increase in the specific surface area of the adsorbent follows an increase in the number of sites available for CTCPFs^23^. The absorption of Cd (II) removal percentage rose from 81.79 to 99.84% when the adsorbent dosage was increased from 0.1 g/250 ml to 1 g/250 ml, according to the results^24^ (Fig. 10b).

### Effect of temperature

**Fig. 11:**
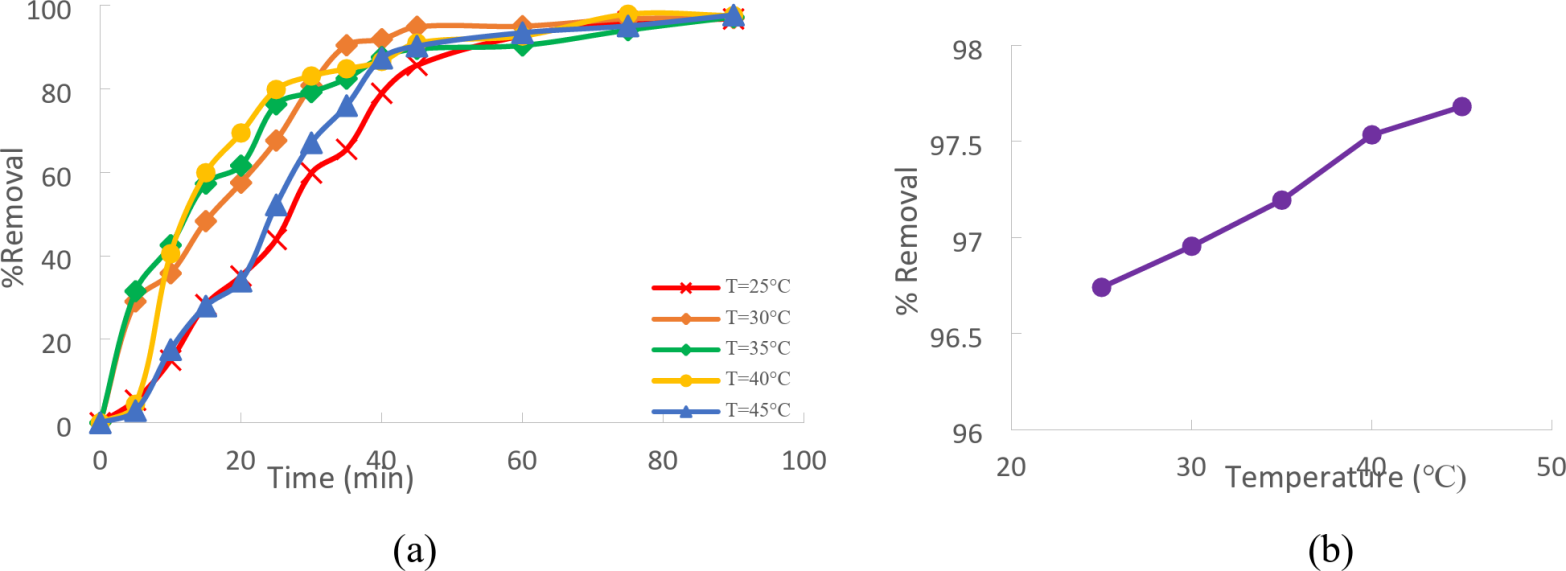
(**a**) Effect of Temperature on % removal of Cd (II) onto CTCPFs, Cd (II) C_0_ = 50 mg/L, CTCPFs dose: 1 g\250 ml stirring speed = 300 rpm, t = 90 min and pH = 5. (**b**) Effect of temperature on Cd (II) removal.

The effect of temperature on the % of Cd (II) removed from wastewater using CTCPFs is depicted in (Fig. 11a). Temperature ranges between 25, 30, 35, 40, and 45 °C were used in the study, along with Cd (II) C_0_ = 50 mg/L and 1 g/250 ml for CTCPFs dosage. The result showed that raising the temperature in just ninety minutes enhanced the removal efficacy. Metal ion mobilities rise with temperature while their mutual retarding forces diminish. Adsorption capacity, the chemical interaction between the adsorbent and adsorbate, and the formation of active surface centers are all further increased by improving intra-particle Cd (II) ion diffusion of the adsorbent’s pores at a higher temperature. The adsorption process appears to be endothermic based on the observed increase. The promotion of the metal-ion interaction, an increase in the mobility of the metal ions and adsorbent active sites, and a decrease in the liquid’s viscosity are some of the possible causes of this enhancement in Cd (II) uptake as temperature rises.

Furthermore, Cd (II) diffuses from the Solution to the adsorbent surface more quickly at higher temperatures. This is in line with the endothermic nature of the adsorption process^25^. The removal efficiency increased with temperature because higher temperatures encouraged adsorption at the Cd(II) adsorbent’s coordination sites, making removing Cd(II) easier. This resulted from some previously slow stages being sped up and new activation sites being added to the adsorbent surface^26^. As the temperature rises to 45 °C, the results show that the uptake of Cd (II) removal increases to 96.68% (Fig. 11b).

## Adsorption of kinetics

### Pseudo–first order model

Most likely, the first known example of a rate of adsorption description is the pseudo-first-order. (Lagergren, 1898) proposed a pseudo-first-order kinetic model, which is represented by Eq. 4:

4 $$\:\text{l}\text{n}\:\left({\text{q}}_{\text{e}}-{\text{q}}_{\text{t}}\right)=\text{l}\text{n}\left({\text{q}}_{\text{e}}\right)-{\text{k}}_{1}\text{t}\:$$

Equation (4) at t = 0, qt = 0 and t = t

5 $$\:{\text{q}}_{\text{t}}=\frac{({\text{C}}_{0}-{\text{C}}_{\text{t}})\times\:\text{v}}{\text{m}}$$

6 $$\:{\text{q}}_{\text{e}}=\frac{({\text{C}}_{0}-{\text{C}}_{\text{e}})\times\:\text{v}}{\text{m}}$$

**Fig. 12:**
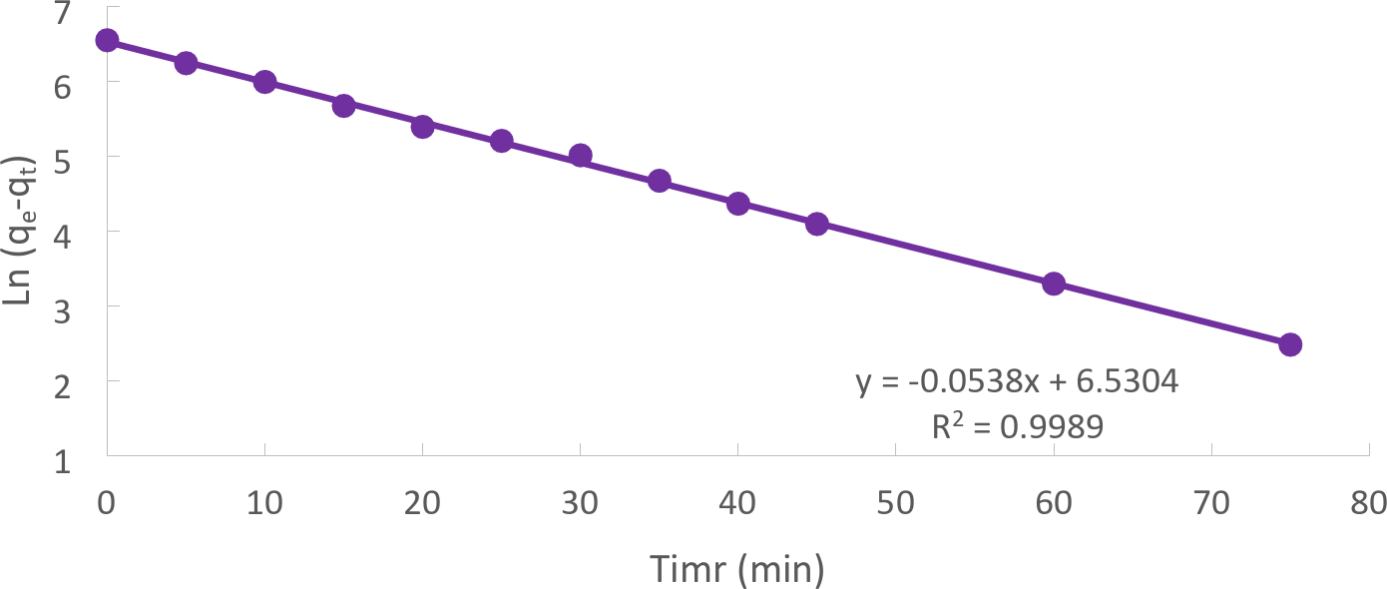
Pseudo-first order kinetic for Cd (II)plots for the adsorption onto CTCPFs at (initial concentration = 50 mg/L, CTCPFs dose = 0.5 g/250 ml, stirring speed = 300 rpm T = 301 K and time = 90 min, pH = 5).

The linear link that results from plotting ln (q_e_-q_t_) against t Fig. 12 allows for the estimation of k_1_ and q_e_, respectively, based on the graph’s slope and intercept^27^.

### Pseudo–second order model

Equation of second-order kinetics:

7 $$\:\frac{{\text{d}\text{q}}_{\text{t}}}{\text{d}\text{t}}={\text{k}}_{2}{\left({\text{q}}_{\text{e}}-{\text{q}}_{\text{t}}\right)}^{2}\:$$

Where the second-order adsorption rate constant is denoted by k_2_. (mg g^-1^min^-1^). Equation (7) is produced by separating the variables and integrating them at the conditions (q_t_=0 at t = 0 and q_t_=q_t_ at t = t). Equation (8) can also be used to describe the adsorption kinetics.

The model’s linearized integral form:

8 $$\:\frac{\text{t}}{{\text{q}}_{\text{t}}}=\frac{1}{{\text{k}}_{2}{\text{q}}_{\text{e}}^{2}}+\frac{1}{{\text{q}}_{\text{e}}}\text{t}\:$$

**Fig. 13:**
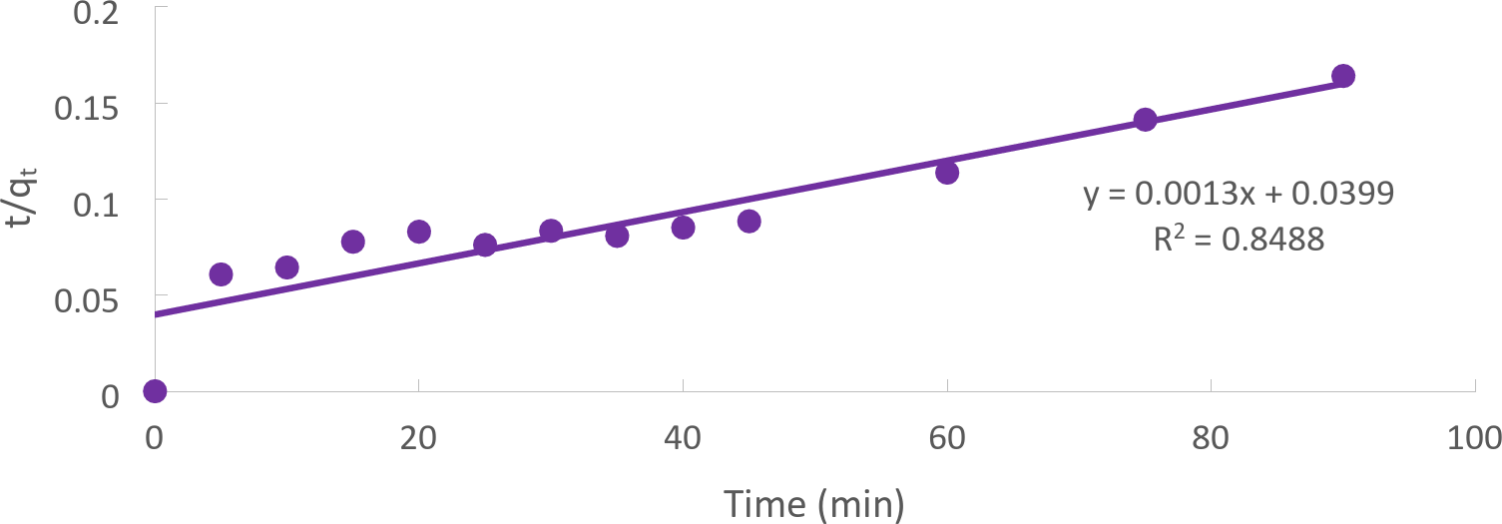
Pseudo-second order kinetic plots for Cd (II) plots for the adsorption onto CTCPFs at (initial concentration = 50 mg/L, CTCPFs dose = 0.5 g/250 ml, stirring speed = 300 rpm T = 301 K and time = 90 min, pH = 5).

According to Eq. (8)’s integral form, there should be a linear relationship between time and the amount of Cd (II) adsorbed (t/q_t_). The relationship between the ratio of time/adsorbed amount of Cd (II) (t/q_t_) in our case of Cd (II) adsorption is a linear function of time (Fig. 13).

Because the pseudo-first-order kinetic model’s correlation coefficient (R^2^) is larger than the pseudo-second-order kinetic model’s, the results showed that physical adsorption regulates the total Cd (II) adsorption rates^28^.

### Weber and Morris model

The Weber and Morris or intra-particle diffusion model is of major relevance because, in most liquid systems, internal diffusion regulates the adsorption rate. A general example of kinetics is given by Eq. (9), where the intercept is connected to mass transfer over the boundary layer, and the predicted value of the exponent is 0.5.

9 $$\:{q}_{t}=\:{K}_{m}{t}^{0.5}+C$$

The constant for intraparticle diffusion rate (mg/g min^0.5^) is represented by k_m_.

**Fig. 14:**
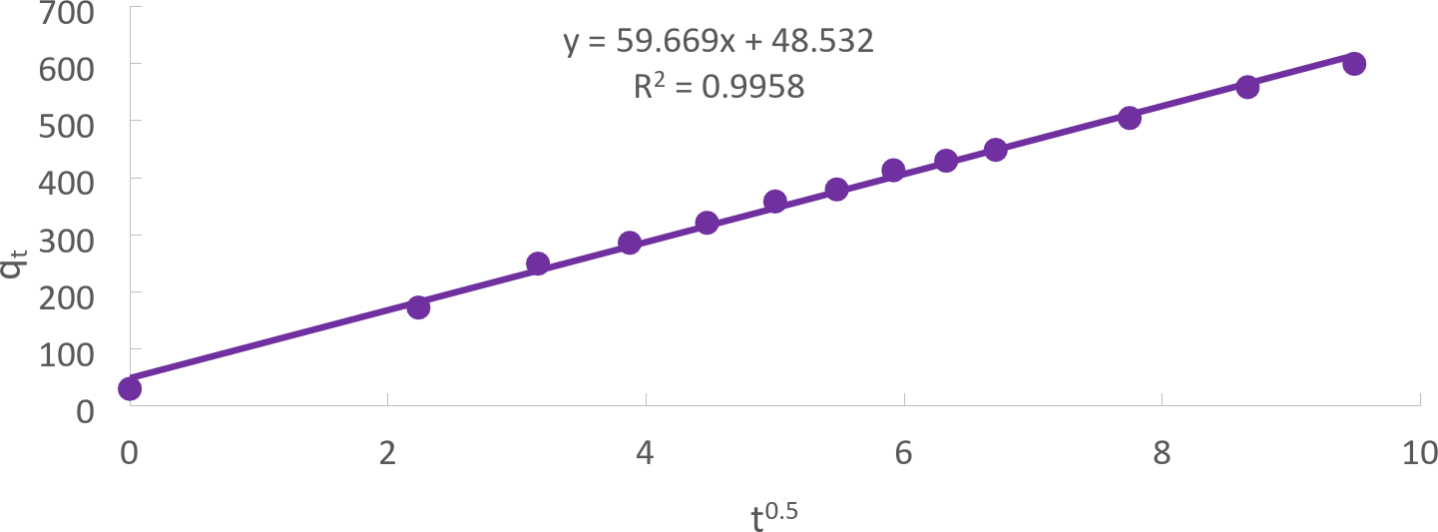
The Weber and Morris model for Cd(II) adsorption onto CTCPFs at initial concentration = 50 mg/L, CTCPFs dose = 0.5 g/250 ml, stirring speed = 300 rpm T = 301 K and time = 90 min, pH = 5).

A straight line with a km slope and an intercept of C is produced when plotting qt against t^0.5^, suggesting that intra-particle diffusion is involved in adsorption. The boundary layer’s thickness can be roughly estimated using the values of C; the thicker the boundary layer, the higher the C value^29^ (Fig. 14).

The Kinetic model’s parameters for Cd (II) adsorption onto CTCPFs are computed in Table 1 for initial concentrations of 50 mg/L, contact time of 90 min, 300 rpm, and 25 °C.

**Table 1:** The kinetic model’s parameters for cd (II) adsorption.

Kinetic models	Parameters	Weight of adsorbent = 0.5 g
Pseudo 1st order equation	q_e_ (EXP.) (mg/g)	685.7
	q_e_ (calc.)	599.8
	R^2^	0.9989
	K_1_(min^− 1^)	53.8
Pseudo 2nd order equation	q_e_ (calc.)	76.9
	R^2^	599.8
	K_2_ (g/mg.min)	0.8488
Weber and Morris model	C	$\:4.24\times\:{10}^{-3}$
	R^2^	48.532
	K_m_ (mg g^− 1^min^− 1^)	0.9932

The Weber and Morris model is useful in identifying if intra-particle diffusion plays a significant role in the adsorption of Cd^+ 2^ onto CTCPFs. the plot is linear and the correlation coefficient (R^2^ ) is high, then the adsorption process is likely governed by intra-particle diffusion.The analysis of K_m_ and C helps understand the dynamics of mass transfer and boundary layer effects in the adsorption process.

### Thermodynamic parameters

The standard Gibbs free energy was calculated using the standard enthalpy change (ΔH°), standard entropy change (ΔS°), and adsorption equilibrium data at various temperatures (ΔG°). To calculate the standard Gibbs free energy (ΔG°) of Methylene Blue adsorption, use Eq. (10).

10 $$\:\varDelta\:{\text{G}}^{0}=-\text{R}\text{T}\text{l}\text{n}{\text{K}}_{\text{e}}\:$$

Equation (11) was used to determine the adsorption equilibrium constant, Ke, at each temperature.

11 $$\:{\text{K}}_{\text{e}}=\frac{{\text{q}}_{\text{e}}}{{\text{C}}_{\text{e}}}\:$$

Where T is the absolute temperature, *R* = 8.314 (j/mol. K) is the gas constant, C_e_(mg/l) is the equilibrium concentration of Cd (II) in the Solution, and q_e_ (mg/g) is the amount of Cd (II) ad-sorbed from the Solution at equilibrium.

12 $$\:\text{L}\text{n}\:{\text{K}}_{\text{e}}=\:-\left(\frac{{\varDelta\:\text{H}}^{0}}{\text{R}\text{T}}\right)+\left(\frac{{\varDelta\:\text{S}}^{0}}{\text{R}}\right)$$

**Fig. 15:**
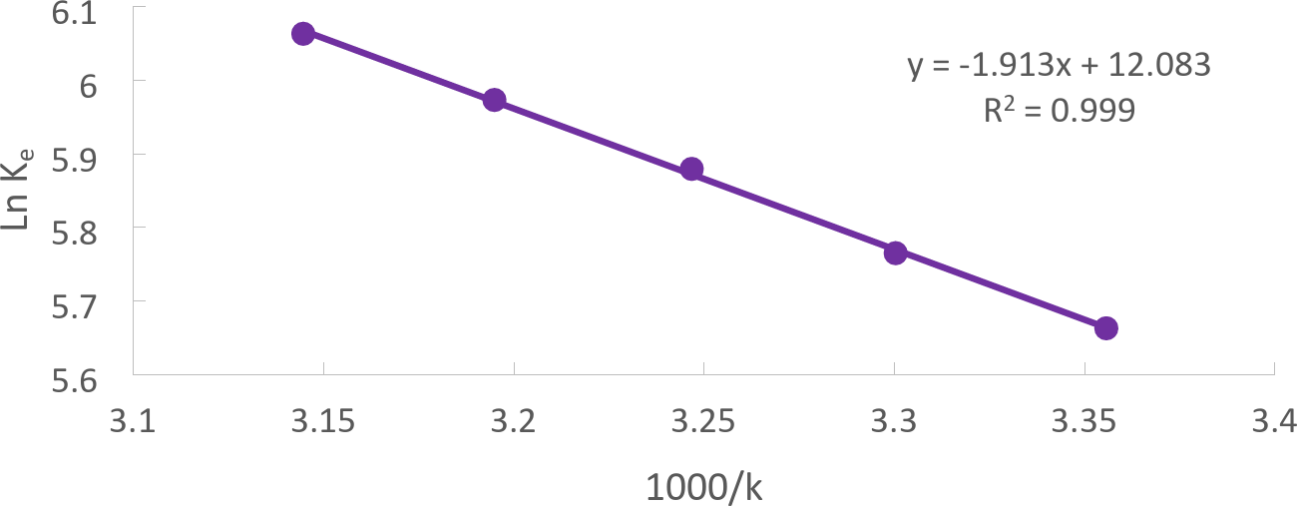
Van̕ t Hoff̓ s plot of adsorption equilibrium constant K_e_ for Solution of initial Cd (II)concentration 50 mg/L at pH = 5, CTCPFs dose 0.5 g /250 ml at 300 rpm, contact time 90 min at different temperatures.

Equation (12), as illustrated in Fig. 15, was used to get ∆H˚ and ΔS° from the slope and intercept, respectively, of the van̕ t Hoff̕ s plot of ln (K_e_) vs. 1/K (Fig. 15).

ΔH° shows the quantity of energy required for this process. The fact that ΔH˚ is positive indicates that a considerable amount of energy is needed to exchange Cd (II)ions.

ΔS° provides information about the system’s unpredictability. The positive value of ΔS° in this instance indicates the presence of both an adsorbent (the substance that takes out the Cd (II) ions) and an adsorbate (the Cd (II) ions) in the system.

ΔG° shows the feasibility and spontaneity of the adsorption processes. In this instance, the negative value of ΔG° indicates that adsorption processes are feasible and occur naturally, i.e., without requiring an energy input from outside sources.

The exchange of the divalent Cd (II) demands a substantial amount of energy, as evidenced by the positive value of ∆H°, according to the values of ΔG°, ∆H°, and ΔS° obtained from Table 2. Moreover, a positive value of ΔS° indicates that the system contains both an adsorbent and an adsorbate. Finally, the fact that ΔG° is negative means that adsorption processes are both possible and spontaneous^30,31^.

**Table 2:** Thermodynamic parameters.

Cd (II)removal by CTCPFs (C_o_ =50 mg/L)	T (K)	298	303	308	313	318
	1000/T (K^− 1^)	3.36	3.30	3.25	3.19	3.14
	q_e_	530.91	576.157	477.51363	507.9767	589.758
	C_e_	1.789	1.81	1.378527	1.28423	1.4
	K_e_	296.8	318.32	346.4	395.55	421. 3
	lnK_e_	5.67	5.77	5.88	5.97	6.06
	ΔG° (kJ/mol)	− 14030.3	− 14,523	− 15055.91	− 15544.15	− 16030.31
	∆H˚ (KJ/mol)	15.9				
	ΔS° (KJ/mol. K)	100.02				

### Isothermal models

The critical step in determining the maximum capacity of adsorbents is the analysis of equilibrium data. It is essential to figure out the adsorbents’ maximum capacity. It is crucial to create an equation that can be used to design and precisely represent the outcomes. The two equations that are most frequently employed for simulating the equilibrium of an adsorption system are the Freundlich and the Langmuir equations.

### Langmuir adsorption isotherm

According to the Langmuir model, Cd (II) is absorbed by monolayer adsorption on a homogenous surface, with no interaction between sorbed species. The Langmuir Eq. (13) is expressed as follows:

13 $$\:\frac{{\text{C}}_{\text{e}}}{{\text{q}}_{\text{e}}}=\frac{1}{{\text{q}}_{\text{m}\text{a}\text{x}}\times\:\text{b}}+\frac{{\text{C}}_{\text{e}}}{{\text{q}}_{\text{m}\text{a}\text{x}}}\:\:$$

Where b is the Langmuir constant, q_e_ is the equilibrium Cd (II) concentration on the adsorbent (mg/L), C_e_ is the equilibrium Cd (II) in the Solution, and q_max_ is the adsorbent’s monolayer adsorption saturation capacity.

**Fig. 16:**
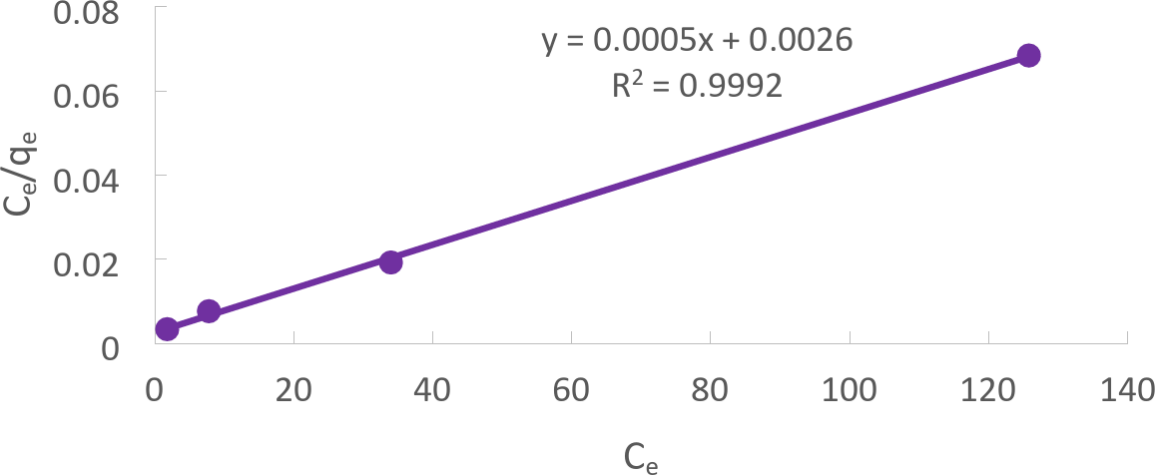
‘s linear plot demonstrates how the adsorption validates the Langmuir adsorption constant and the Langmuir isotherm.

As seen in Fig. 16, a plot of C_e_/q_e_ vs. C_e_ yields a straight line with the values of slope (1/q_max_) and intercept (1/q_max_) at the initial concentration of Cd (II).

Figure 16. Langmuir isotherm for Cd (II) adsorption for Solution of initial Cd (II) concentration = 50 mg/L at pH 5, CTCPFs dose 0.5 g /250 ml at 300 rpm, contact time 90 min at temperature = 25℃.

### Freundlich adsorption isotherm

Freundlich adsorption isotherm. At many concentrations, one of the most widely used mathematical models frequently fits the experimental data quite well. This isotherm expresses the exponential distribution of the energy and surface heterogeneity of active sites. The nonlinear form of the Freundlich model is expressed as:

14 $$\:{\text{q}}_{\text{e}}={\text{K}}_{\text{f}}\:{\left({\text{C}}_{\text{e}}\right)}^{\raisebox{1ex}{$1$}\!\left/\:\!\raisebox{-1ex}{$\text{n}$}\right.}\:\:$$

The Freundlich model can be represented in its linear version as follows:

15 $$\:\text{log}{q}_{e}=\text{log}{K}_{f}+\left(\frac{1}{n}\right)\text{log}{C}_{e}\:$$

**Fig. 17:**
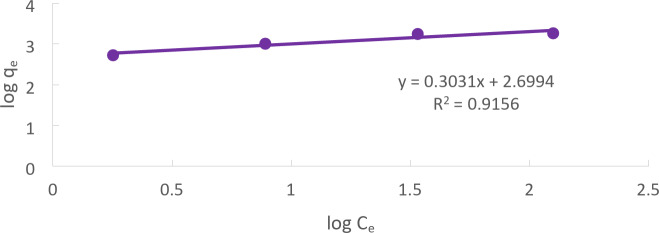
Freundlich Cd (II) adsorption for Solution of initial Cd (II) concentration = 50 mg/L at pH = 5, CTCPFs dose 0.5 g /250 ml at 300 rpm, contact time 90 min at temperature = 25 ℃.

The sorption intensity is represented by the constant n, which varies based on how heterogeneous the adsorbents are, and the adsorption capacity is defined by the Freundlich constant K_F_. A plot of log q_e_ versus log C_e_, as shown in Fig. 17, produces a straight line with a slope of 1/n and an intercept of log K_F_^32^.

The relationship between a substance’s concentration in a solution and the amount that can be adsorbed onto a solid surface is described by the Langmuir and Freundlich isotherm constants.

The greatest amount of the substance that can be adsorbed onto the solid surface per unit mass of the solid is represented by the q_max_(mg/g) value. The affinity or strength of the adsorption process is defined by the b (L/mol) value. A higher b value indicates a stronger affinity between the material and the solid surface.

The R2 value indicates the degree to which the actual data matches the mathematical model. A perfect fit is indicated by a value of 1, whereas a poor fit is indicated by a value of 0.

The Freundlich model parameter 1/n describes the adsorption process’s heterogeneity. A method is homogenous if its value is 1 and more heterogeneous if it is less than 1. The solid surface’s adsorption capability is shown by the Kf(mg/g) value. A higher Kf value indicates a larger adsorption capacity.

According to the data, the Langmuir model is a more accurate model for explaining Cd (II) adsorption onto a solid surface in the particular case of Cd (II) since it fits the experimental data better. There is no further explanation for the specific figures shown in Table 3^33–35^.

**Table 3:** Shows a comparison between Langmuir and Freundlich models.

	Langmuir isotherm constants			Freundlich isotherm Constants		
	q_max_(mg/g)	b (L/mol)	*R* ^2^	1/*n*	K_f_(mg/g)	*R* ^2^
Cd (II)	2000	0.192	0.9992	0.3031	500.5	0.9156

A model known as the Langmuir isotherm explains how molecules absorb onto a solid surface to form monolayers. It is predicated on the idea that absorption takes place in the adsorbent at particular homogenous locations. Once a molecule has taken up residence there (monolayer coverage), no more adsorption can occur at a spot. There is no further explanation for the particular figures shown in Table 3.

Adsorbed molecules do not interact with one another; all sites are equal. The dimensionless separation factor , a crucial component of the Langmuir isotherm, is used to forecast the adsorption process’ favorability and reveal its characteristics. It is also known as the coefficient of equilibrium analysis.

16 $$\:{R}_{l}=\frac{1}{1+{K}_{l}{Q}_{m}}$$

R_l_ is utilized to calculate the adsorption affinity. Favorable adsorption, defined as 0 < R_L_ < 1; unfavorable adsorption, defined as R_L_ > 1; linear adsorption, defined as R_L_ = 1; and irreversible adsorption, defined as RL = 0, are the four possible ways to characterize the constant. The Cd(II) R_L_ value was 0.059, indicating favorable adsorption.

Based on the kinetic and isotherm model fitting results from the previous data, the adsorption mechanism of Cd(II) onto CTCPFs primarily occurs through physical adsorption, as indicated by the pseudo-first-order kinetic model, suggesting adsorption on homogeneous sites without interaction between adsorbed molecules. The Langmuir model supports monolayer adsorption, while the Weber and Morris model indicates that intra-particle diffusion limits the rate of equilibrium. Thermodynamic results show that the process is spontaneous and endothermic. These findings suggest that the adsorption is efficient, driven by the physical transfer of Cd (II) ions from the solution to the adsorbent surface.

## Mathematical modeling of the experimental system

Many tests are required to determine the optimal conditions for adsorption; however, optimization can be achieved simultaneously with fewer experiments by applying statistical experimental design techniques. Therefore, calculating the optimal values of ingredients, creating a mathematical model that shows how each aspect affects the product, and utilizing fewer chemicals in less time, all contribute to significant cost reductions^36^.

### Methodology

The RSM technique is one area of mathematical modeling application that looks at the relationships between different parameters. Dependent variables are defined via the response-specific Modeling (RSM) approach, which uses independent variables as a factor. RSM optimization and Modeling show how different factors impact the Response. The RSM approach is first established by utilizing screening designs to pinpoint the crucial factors affecting the Response. These techniques use various experimental design models, such as the Box Behnken Design, the Central Composite Design, and the Optimal Custom Design. In this work, activated Carbon from a palm frond was utilized to optimize the Cd (II) removal percentage (%) employing Box-Behnken design^37,38^.

### Experimental design

The effects of each independent variable (time (min), concentration (mg/L), and dose (mg)) and optimization can be explained by a mathematical model. The Cd (II) removal percentage (%) is optimized by the box-Behnken design using Chemically Treated Carbon from palm fronds. Consequently, the estimated results are also given in addition to the experimental data^39^.

A BBD with three elements was discovered at three levels (-1, 0, and 1)^40^. Table 4 displays the levels and attributes. The effects of the three independent factors (time (min), A, concentration (mg/L), B, and dose (mg), C) on the percentage of Cd(II) eliminated using CTCPFs were assessed using BBD, and it was shown that 15 experimental runs were necessary. The experimental setup and the CTCPFs’ conclusions on the percentage (%) of Cd (II) eliminated from the aqueous solution are shown in Table 4. Regression analysis of the Answer produced a model, and analysis of variance (ANOVA) and the F test was used to assess its effectiveness^41,42^. The model’s responses were created using polynomial functions, and data analysis and system optimization were carried out using the statistical program “Statistics.” (Calculation 17):

**Table 4:** BBD experimental variables and their levels.

Variable code	Name of variable	levels		
		− 1	0	+ 1
A	Time (min)	0	30	90
B	Concentration (mg/L)	30	150	300
C	Dose (mg)	0.1	0.5	1

17 $$\:\text{Y}={{\upbeta\:}}_{0}+\sum\:{{\upbeta\:}}_{\text{i}}{\text{x}}_{\text{i}}+\sum\:{{\upbeta\:}}_{\text{i}\text{i}}{\text{x}}_{\text{i}2}+\sum\:{{\upbeta\:}}_{\text{i}\text{j}}{\text{x}}_{\text{i}}{\text{x}}_{\text{j}}$$

Y denotes the expected response, the intercept term is β_0_, the linear effect is β_i_, the square effect is βii, and the interaction effect is β_ij_.

### RSM analysis for optimization of cd (II) removal %

In this work, the Cd(II) removal percentage (%) by activated Carbon from palm frond for RSM optimization was calculated using several parameters, including pH, concentration (ppm), and dose (mg). After fifteen trials, the BBD method—which is used to optimize experimental data—was finished. Consequently, Table 5 presents the theoretical and experimental results achieved with the design experimental circumstances. Provides the Y (Cd(II) elimination percentage (%)) value in the quadratic model below based on the obtained results. The interaction parameters in this Equation were X_1_ × _2_, X_1_ × _3_, and X_2_ × _3_. The linear parameters in this Equation were X_1_, X_2_, and X_3_. The second-order parameters in the model were X_12_, X_22_, and X_32_^43^.

**Table 5:** BBD for Cd(II) removal percentage (%)using chemically treated carbon of palm frond.

Trail	pH	Concentration of Cd(II) (ppm)	Adsorbent dose (gm)	Cd(II) removal percent (%) from aqueous solution using chemically treated carbon of palm fronds	
				Measured	Predicted
1	5	50	1	99.84	100.0883
2	5	150	0.5	88.85	89.93318
3	8	150	1	74.61	75.954
4	5	150	0.5	88.85	89.93318
5	8	50	0.5	78.61	78.27198
6	5	50	0.1	81.97	82.63258
7	8	150	0.1	56.61	57.69386
8	2	300	0.5	54.29	58.95668
9	5	150	0.5	88.85	89.93318
10	2	50	0.5	90.29	90.21918
11	8	300	0.5	42.61	47.15948
12	5	300	0.1	45.97	49.99508
13	5	300	1	66.84	70.7133
14	2	150	1	87.29	88.3974
15	2	150	0.1	68.34	69.1361

18 $$\:Y\%=54.0168+11.6105{X}_{1}+0.007{X}_{2}+52.693{X}_{3}-1.3514{X}_{1}^{2}-0.0004{X}_{2}^{2}-30.0875{X}_{3}^{2}+0.0001{X}_{1}{X}_{2}-0.1854{X}_{1}{X}_{3}+0.0142{X}_{2}{X}_{3}$$

In this case, Y(%) is the proportion (%) of Cd(II) removed from the palm frond by activated Carbon. X_1_ denotes pH, X_2_ concentration (ppm), and X_3_ dose (mg).

BBD in a conventional behavior was the foundation for the adsorption processes that the RSM investigation examined. Nearly all single-parameter tests use activated Carbon from palm fronds to investigate the levels of variables like pH, concentration (ppm), and dose (mg) that affected the Cd(II) removal percentage (%). The benefit of RSM is that fewer experiments are needed, which lowers the cost of more sophisticated analysis techniques. BBD or CCD can be used for this strategy. Activated Carbon from the palm frond was used in this work to optimize Cd(II) removal percentage (%) using BB) with various parameters^44^. Experiments were carried out after deciding on the parameter levels shown in Table 5.

The standard deviation of the data from the mean value is represented by the parameter F-value in Table 6, which displays the results of statistical analysis of variance (ANOVA). Generally, when the F-value is very high and the Value of “prob > F” is less than 0.05, the model is statistically significant at the 95% confidence limit and accurately predicts test results. Model terms are not important when their values are more extensive than 0.1000. The F-value and p-value for this model were 17.74493 and 0.000159, respectively, showing that the activated Carbon from the palm frond used was fully significant^45,46^.

**Table 6:** ANOVA results for cd (II) removal percentage (%) using chemically treated carbon of palm frond.

Source	Sum of Square	df	Mean of square	F-value	*p*-value	Significant or insignificant model terms
Model	3647.048	3	1215.683	17.74493	0.000159	Significant

The compatibility of the experimental data with the theoretical values according to the model is demonstrated by the values of R2 (0.999), which is a measure of variation compatibility of the model for Cd(II) removal percentage (%), and R^2^ adj (0.9992), which represents the corrected terms and is a measure of the amount of change in the model. These values are obtained using the RSM method^47^.

### Three-dimensional (3 D) response surface graphs response surface plots for RSM

**Fig. 18:**
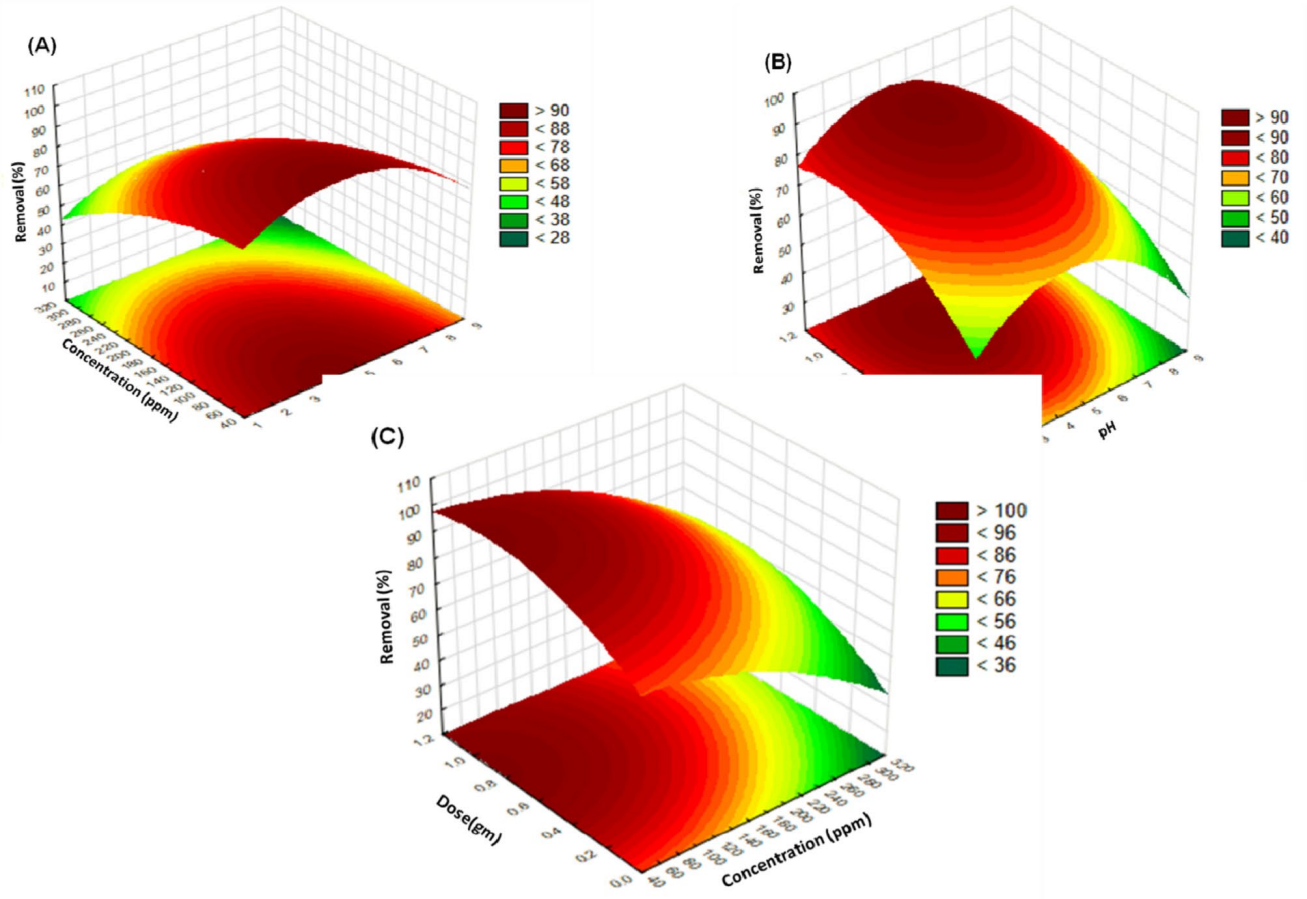
Response surface plots for Cd(II) removal % using Chemically Treated carbon of palm frond: (**A**) Effect of Concentration/pH [at Dose: 0.5gm; at 25 °C]; (**B**)effect of Dose/pH [at Cd(II) concentration = 50 ppm; at 25 °C]; (**C**) effect of Dose/Concentration at[time:90 min; at 25 °C].

Figure 18 displays three-dimensional surface graphs of the parameters influencing the percent (%) of Cd (II) removal according to RSM. To determine the significant effects of interactions together with the various variables on the Cd (II) removal percent (%) utilizing activated Carbon from Raphanus seeds residual, the predictor variables (Fig. 18A-C) were plotted on the X and Y axes and response on the Z axis. For each pairwise combination of the three components (X_1_ × _2_, X_1_ × _3_, X_2_ × _3_), the response surface in three dimensions and its corresponding two-dimensional contour plot were created, with the third factor remaining at its center point level (0). The dose and concentration interaction effect produced the most significant reaction, as seen in Fig. 18C.

Figure 18 (A, B) shows the effects of pH, concentration, and dose, respectively, on the percent (%) of Cd(II) removed using chemically treated Carbon of palm fronds. The percentage of Cd(II) removed increased with time until it reached 5, after which it decreased from 5 to 8. These surface-active sites become more negatively charged as pH rises, which reduces competition and increases the electrostatic attraction force that can facilitate the adsorption of positively charged metal ions.

Meanwhile, protonation of the cell wall component negatively impacted the modified adsorbent capacity for biosorption at an acidic pH (pH ≈ 3), and the impact lessened as the medium’s pH increased. This is because there is more competition. It has been suggested that carboxyl groups are in charge of metal binding. It is well known that the carboxyl group ionizes more frequently at pH levels higher than 4.

Similar to when Cd(II) ions biosorbent at a pH more than 6.0, at pH ≥ 7.0, hydroxy species of the metal ion that are not bonded to the functional surface may start to develop in the system. This would have resulted in a decrease in the biosorbent’s Cd(II) removal percentage (%) for the biosorption of Cd(II) ions^48^.

With Chemically Treated Carbon of palm fronds, Fig. 18 (B, C) shows the effect of dose and pH concentration, respectively, on Cd(II) removal percent (%). It can be seen that Cd(II) removal percent (%) increases as the dose increases from 0.1 to 1 gm until reaching equilibrium at 0.5 gm because the Cd(II) solution contains a fixed amount of Cd(II) molecules and the adsorbent’s surface area increases because more active sites are available over the adsorbent surface^45–49^.

The effects of Cd(II) concentration and pH dose, respectively, on the percent (%) elimination of Cd(II) using activated Carbon from residual Raphanus seeds are displayed in Fig. 18 (A, C). The maximum Cd(II) removal percentage (%) was achieved using 50 ppm of Cd(II) because a higher initial concentration of Cd (II) ions increased the driving force to overcome the mass transfer resistance of Cd(II) ions between the aqueous and solid phases, increasing the likelihood of a collision. It is observed that Cd(II) removal percent (%) decreases as Cd(II) concentration increases from 50 to 300 ppm. The relationship between palm frond activated carbon and Cd(II) ions likewise declined with Cd(II) removal percentage (%).

## Conclusion

We investigated the sorption of Cd (II) from an aqueous solution in our work. This study used organically generated activated Carbon that was made from palm leaves as the adsorbent. H_3_PO_4_ was applied to the activated Carbon to improve its characteristics. Finding an adsorbent that is not only efficient but also reasonably priced and readily available was the major goal of our research. We used the batch equilibrium technique to do this. The results of the investigation show that the adsorbent’s capacity to absorb Cd (II) is dependent on the concentration, dosage, and pH of the original Solution With Chemically Treated Carbon of palm fronds with H_3_PO_4_ had an adsorption capacity of 99.96%. The positive value of ∆H˞ suggests that a significant amount of energy is needed for the exchange of divalent Cd (II), as demonstrated by the values of ΔG°, ∆H^, and ΔS° obtained from Table 2. Moreover, the positive value of ΔS° indicates that the system contains both an adsorbent and an adsorbate. Finally, the fact that ΔG° is negative means that the adsorption processes are both possible and spontaneous.

After a careful analysis of the sequence’s kinetics and isotherm models, it was found that the experimental results showed a remarkable fit to both the Freundlich and Langmuir models. However, after more investigation, it was discovered that the adsorbent’s identified Langmuir and Freundlich isotherm features unmistakably demonstrated the advantageous nature of the adsorption process. Therefore, it can be inferred from these results that chemically treated Carbon made of palm fronds treated with H_3_PO_4_ has a comparatively higher adsorption efficiency, making it appropriate for removing Cd (II) from aqueous solutions.

## Data Availability

All data find in the manuscript.
